# Application of Bioinformatics in Predicting the Efficacy of Digestive Tumour Immunotherapy Target TIM-3 and its Inhibitors

**DOI:** 10.7150/jca.92446

**Published:** 2024-02-12

**Authors:** Zexin Wang, Xibin Li, Litao Tian, Dan Sha, Qinhui Sun, Jinshen Wang

**Affiliations:** 1 Department of Gastrointestinal Surgery, Shandong Provincial Hospital Affiliated to Shandong First Medical University, Jinan, Shandong 250021.; 2 Department of Minimally Invasive Treatment of Cancer, Shandong Provincial Hospital Affiliated to Shandong First Medical University, Jinan, Shandong 250021.

**Keywords:** Digestive tumours, TIM-3, GEO, pathway and Immunotherapy

## Abstract

**Background:** Our main objective is to apply bioinformatics in predicting the efficacy of digestive tumour immunotherapy target TIM-3 and its inhibitors.

**Methods:** Our study used the gene expression omnibus (GEO) database to identify datasets associated with digestive tumours and the action of TIM-3. The GSE427729 dataset based on the GPL10192 platform. The dataset consisted of six samples of total RNA derived from TIM-3 control and knockdown RAW 264.7 cells. We used GEO2R tool to identify DEGs before performing Gene Ontology and identifying the kyoto encyclopedia of genes and genomes (KEGG) pathways. Lastly, we determined the PPI networks to identify hub genes.

**Results:** Our study identified 57 differentially expressed genes based on an adjusted p-value of less than 0.05 and a log2 fold change of 2.0. There were 26 down-regulated genes with 31 up-regulated genes while 22, 404 genes were non-significant. The DEGs were enriched in biological pathways such as activating leukocytes, cells, and development of the immune system. Additionally, we identified four significant KEGG pathways that were implicated in digestive tumour immunotherapy and TIM-3; pathways of pancreatic cancer, NF-Kappa B signalling pathway, Toll-like receptor signalling pathway and C-type lectin receptor signalling pathway. The PPI networks identified 10 hub genes that were implicated in digestive tumour immunotherapy target TIM-3 (Myd88, Traf6, Irf7, Cdk4, Ccnd2, Mapkap1, Prr5, Mpp3, Serpinb6b and Pvrl3).

**Conclusion:** Targeting these biological pathways, KEGG pathways, molecular functions and cellular processes can lead to novel therapeutic treatment and management in digestive tumours based on TIM-3 immunotherapy.

## Introduction

Gastric cancer has one of the greatest mortality rates worldwide and is often characterised by poor prognosis 1. In 2022, more than one million cases of gastric cancer were diagnosed and reported worldwide 2. Analysis from the global cancer estimates proposes that millions of cancer patient are diagnosed and reported annually despite the presence of advanced techniques such as chemotherapy, radiotherapy, immunotherapy or molecular therapy 3. These advanced treatment techniques have minimal effects in improving the 5-year survival rate; therefore, more research is required to identify and develop novel therapeutic techniques in the treatment and management of cancer.

According to Rafii et al. 4, the identification and development of techniques that adopt biomarkers is essential in the treatment and management of tumours. Immunotherapy is a critical approach in the management of tumours and can be designed for effective tumour metastasis and progression of tumours 5, 6. Immunotherapy has a higher success rate in inhibiting the growth and development of malignant tumours. For instance, the development of PD-1 and PD-L1 have revolutionized biomarkers in the treatment of cancer because they can be incorporated into the diagnosis of several cancers. Moreover, prognostic biomarkers such as cytotoxic T lymphocyte antigen-4 (CTLA-4) and PD-1 are utilised in immunotherapy with a clinical success rate of over 80%.

Phong et al. 7 suggested that the T-cell immunoglobulin and mucin -dominant containing-3 (TIM-3) is a significant immune checkpoint molecule in the diagnosis and treatment of gastric cancers. Similarly, Das et al. 8 proposed that TIM-3 has a critical role in suppressing the action of cytotoxic lymphocytes and response of Th1 cells. Moreover, it has immunosuppression action on inflammatory cytokines such as interferon-gamma, interleukins or tumour necrosis alpha. Solinas et al. 9 proposed that TIM-3 has been implicated in innate and adaptive immune responses by positive or negative regulation. The expression of TIM-3 in cancer cells has been correlated with poor prognosis in gastric cancer, oral squamous cell carcinoma and ovarian cancer 10-12. A study by Duan et al. 13 found non-significant correlations between TIM-3 and survival rates in patients of gullet squamous cell carcinoma. Similarly, a study by Hong et al. 14 found a significant correlation between poor survival outcomes in gullet squamous cell carcinoma and expression of TIM-3.

Du et al. 15 proposed that TIM-3 and immune cells such as NF-kB or CD47 have an essential role in aiding the immune escape of cancer cells. The action of TIM-3 is understood as a co-inhibitor that is expressed in tumour cells, immune cells and other cells. The expression of TIM-3 has a negative regulation effect on immune cells such as CD8 positive T cells or CD4 positive Th1 cells. Therefore, TIM-3 has a regulation effect on the depletion of T-cells and immunosuppression of dysfunctional cells in the growth and development of tumours^16^-^19^. Hong et al. 14 proposed that overexpression of TIM-3 in cancer cells is an indicator of poor prognosis; furthermore, Shan et al. 20 suggested that increased expression of TIM-3 in cancer cell lines can lead to metastasis in the oesophagus and enhance malignant tumours via the AKT signalling pathway.

Das et al. 8 established that TIM-3 is a transmembrane protein that is associated with the Tim family of genes and genomes. The locus of TIM-3 and its genomic family plays a crucial role in allergic and autoimmune infections 21. A dysregulation in the expression levels of TIM-3 leads to progression of autoimmune infections and cancer. Hakemi et al. 22 proposed that TIM-3 has numerous ligands such as Gal-9, CEACAM1 and PtdSer that are expressed in immune tissues and secreted by endothelial cells. These ligands have a critical role in the controlling the levels of inflammation, immune response to tumours, autoimmune response and apoptosis of Th1 cells.

Our study seeks to use apply bioinformatics in predicting the efficacy of digestive tumour immunotherapy target tim-3 and its inhibitors. The incorporation of bioinformatics in the analysis of genes increases the possibility of identifying hub genes susceptible to the infection, molecular pathways, and cellular processes. Our study adopted the Gene Expression Omnibus (GEO) to identify relevant datasets that were screened for differentially expressed genes in TIM-3 and digestive tumours.

## Methods

### GEO Datasets

Our study used the GEO database to identify (https://www.ncbi.nlm.nih.gov/geo/) datasets associated with digestive tumours and the action of TIM-3. In our search, we adopted the following keywords, “TIM-3”, “carcinoma”, “oesophagus squamous carcinoma”, “Gastric Carcinoma”, “hepatocellular carcinoma”, “pancreatic carcinoma” and “Gut”. We obtained the GSE427729 dataset based on the GPL10192 platform (NimbleGen Mus musculus MM9 Expression Array (12x135k)). The dataset consisted of six samples of total ribonucleic acid (RNA) derived from TIM-3 control and knockdown RAW 264.7 cells. There were 135,000 probes per chip which were converted into gene symbols.

### Differentially Expressed Genes (DEGs)

Our study acquired the online GEO2R tool (https://www.ncbi.nlm.nih.gov/geo/geo2r/) for the screening and identification of DEGs based on the adjusted p-value of 0.05. During the analysis, we used the Benjamini & Hochberg based on False Discovery Rates, applied a log transformation to the data and we used the Submitter supplied category of platform annotation. We had two experimental conditions of TIM-3 and control. TIM-3 involved knockdown cells that expressed TIM-3 while control group had no expression of TIM-3.

Furthermore, we used a log2 fold change of greater or equivalent to 1 to determine statistically significant genes. All genes without symbols or genes that had double probes and above were eliminated from the analysis. Our study extracted Venn diagrams (https://www.ncbi.nlm.nih.gov/geo/geo2r/) in locating overlapping differential genes and also extracted the corresponding volcano plots and mean difference plots. We used the Express Network Analyst software to extract and analyze the gene matrix files in determining the statistically significantly up-regulated and down-regulated genes. Also, we obtained the heatmaps (https://www.networkanalyst.ca/).

### Gene Ontology and KEGG Pathways

We used the database for annotation, visualization and integrated discovery (DAVID) (https://david.ncifcrf.gov/) to perform all aspects related to functional gene annotation and gene ontology of the hub genes that are differentially expressed. Additionally, we incorporated the Kyoto Encyclopaedia of Genes and Genomes (KEGG) database to determine the critical biological processes, molecular functions and cellular processes of all DEGs (https://www.genome.jp/kegg/pathway.html). During all analyses, we counterchecked to ascertain that all the gene counts were greater than 20 and the rate of false discovery rates were determined using an adjusted p-value of below 0.05. It is essential to perform gene ontology of the genes implicated in TIM-3 and digestive tumours so as to identify the molecular, cellular and biological pathways.

### Protein-Protein Interaction Networks (PPI)

Our study used the Search Tool for the Retrieval of Interacting Gene Database (STRING) (https://string-db.org/). The STRING tool allowed us to construct the network of interactions of the hub genes based on the MCL clustering algorithm. An interaction score of greater or equivalent to 0.8 was used before exporting all the networks into Cytoscape for analysis of their structures and 3D bubbles.

Our analysis in Cytoscape was based on the MCODE (Molecular Complex Detection) in determining and identifying dense regions of the hub genes. We determined that all the statistically significant DEGs were on a k-interaction score of 4, optimum depth of 50 and a cut-off value of 0.3. In Cytoscape, the CytoHubba plugin was deployed in ranking all the nodes in the network and we classified all the DEGs based on their degree, betweenness and measure of centrality.

## Results

We extracted the gene matrix of the GSE427729 dataset based on the GPL10192 platform (NimbleGen Mus musculus MM9 Expression Array (12x135k)). The dataset consisted of six samples of total ribonucleic acid (RNA) derived from TIM-3 control and knockdown RAW 264.7 cells. There were 135,000 probes per chip which we converted into gene symbols. Using Network Express Analyst, we identified 57 differentially expressed genes based on an adjusted p-value of less than 0.05 and a log2 fold change of 2.0. There were 26 down-regulated genes with 31 up-regulated genes while 22, 404 genes were non-significant.

**Figure 1:**
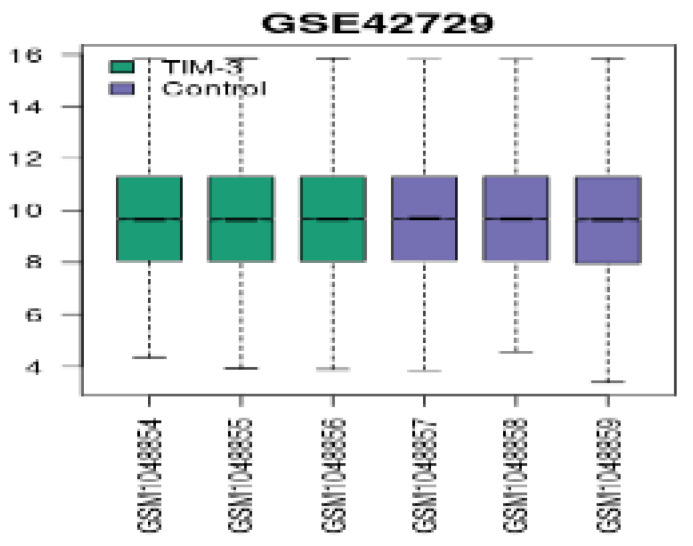
Normalized boxplots of gene expression profiles in GSE42729.

We applied log2 transformation to the data and adjusted for batch effects. The normalized boxplots (refer to Figure 1) show the gene expression profiles in TIM-3 and control groups. All the median values permit cross-comparisons between TIM-3 and control groups; hence, effective normalisation.

### Volcano Plot

**Figure 2:**
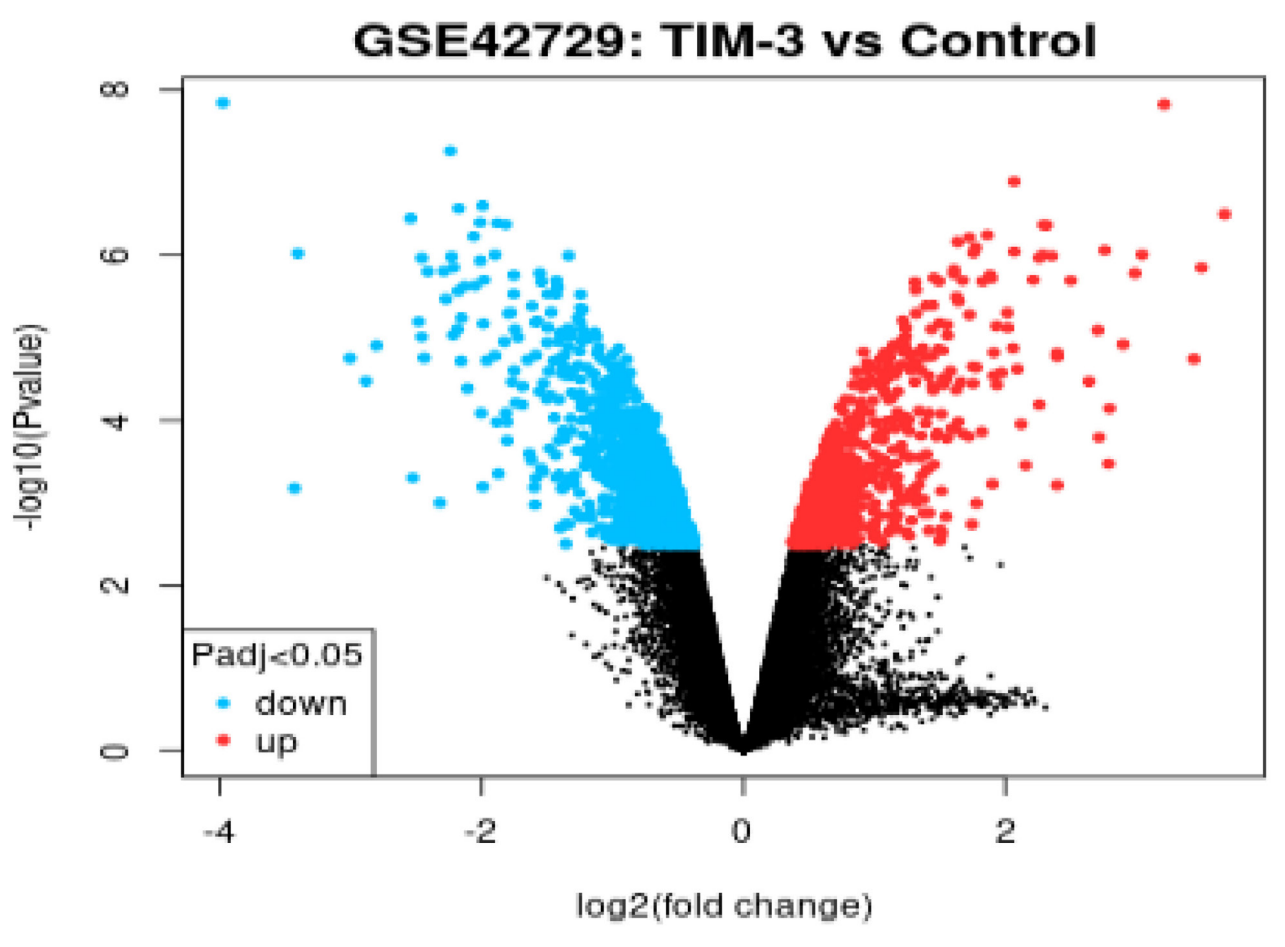
Volcano plot of DEGs.

In reference to Figure 2, we extracted the volcano plot based on adjusted p-values of below 0.05 and a log fold change of greater than 2. The log2 fold change on the x-axis denotes the variation of change and refers to the percentage of expressed gene transcripts to the logarithm of the p-values. All genes in the volcano plot are annotated with the blue section showing down-regulated genes while red showing up-regulated genes. The expression levels of 26 down-regulated genes (blue section) were reduced in TIM-3 compared to the control group. In contrast, the expression levels of 31 up-regulated genes increased in TIM-3 compared to the controls. Th black region represents a total of 22,404 non-significant genes.

### Mean Difference Plot

**Figure 3:**
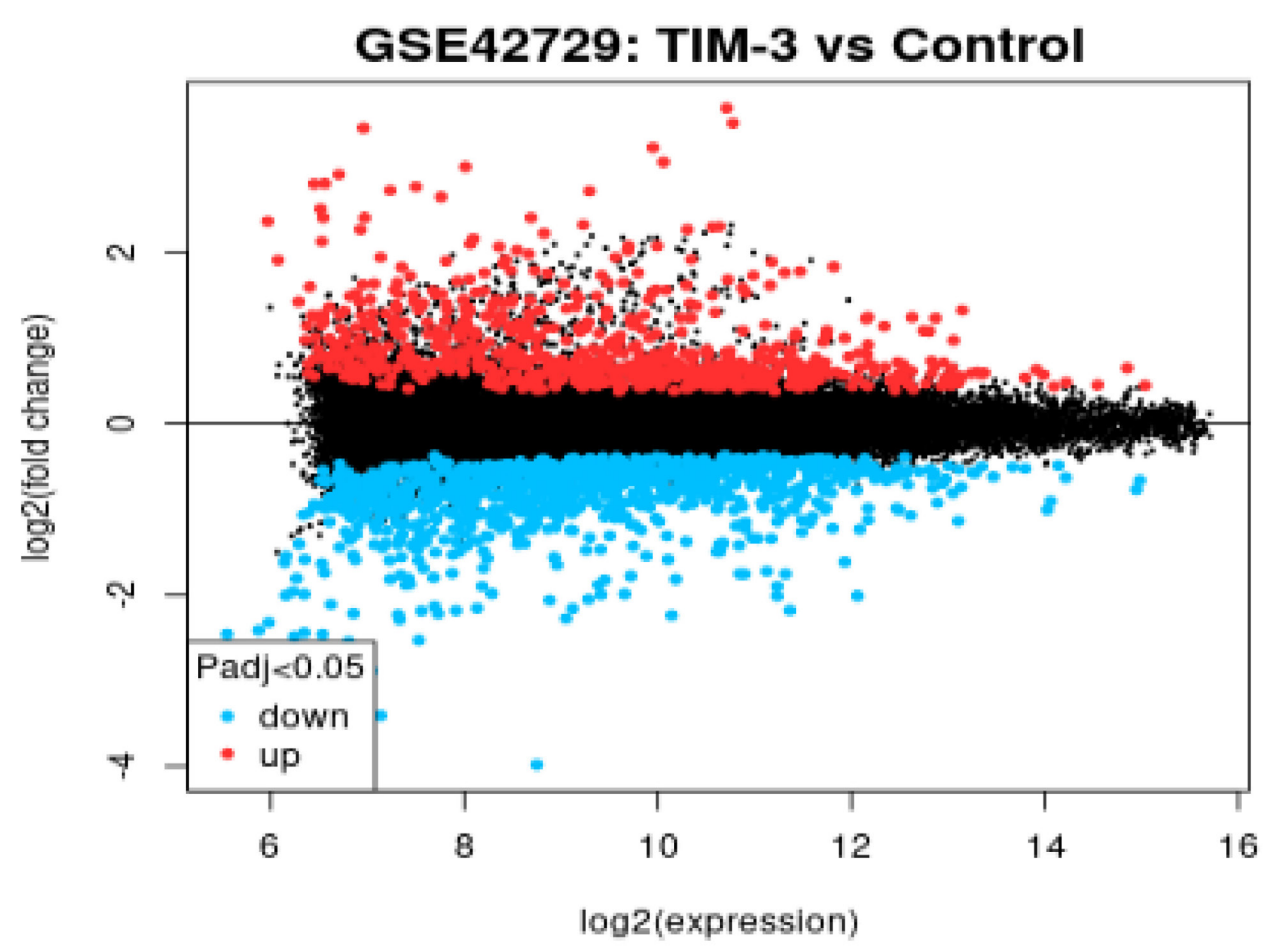
Mean difference plot of DEGs.

In reference to Figure 3, the graph is a visualization of the differences in the intensity ratios of log2 fold change and log2 expression. In this graph, every point is a gene annotated point with the blue section showing down-regulated genes and the red section showing up-regulated genes. The expression levels of these genes vary from the left to the right with genes that are highly expressed occurring on the left side while genes of a lower expression occurring on the right. The expression levels of genes is inversely proportional to the fold change leading to a fanning effect because the genes appear to move from the right hand side to the left section.

### Venn Diagram

**Figure 4:**
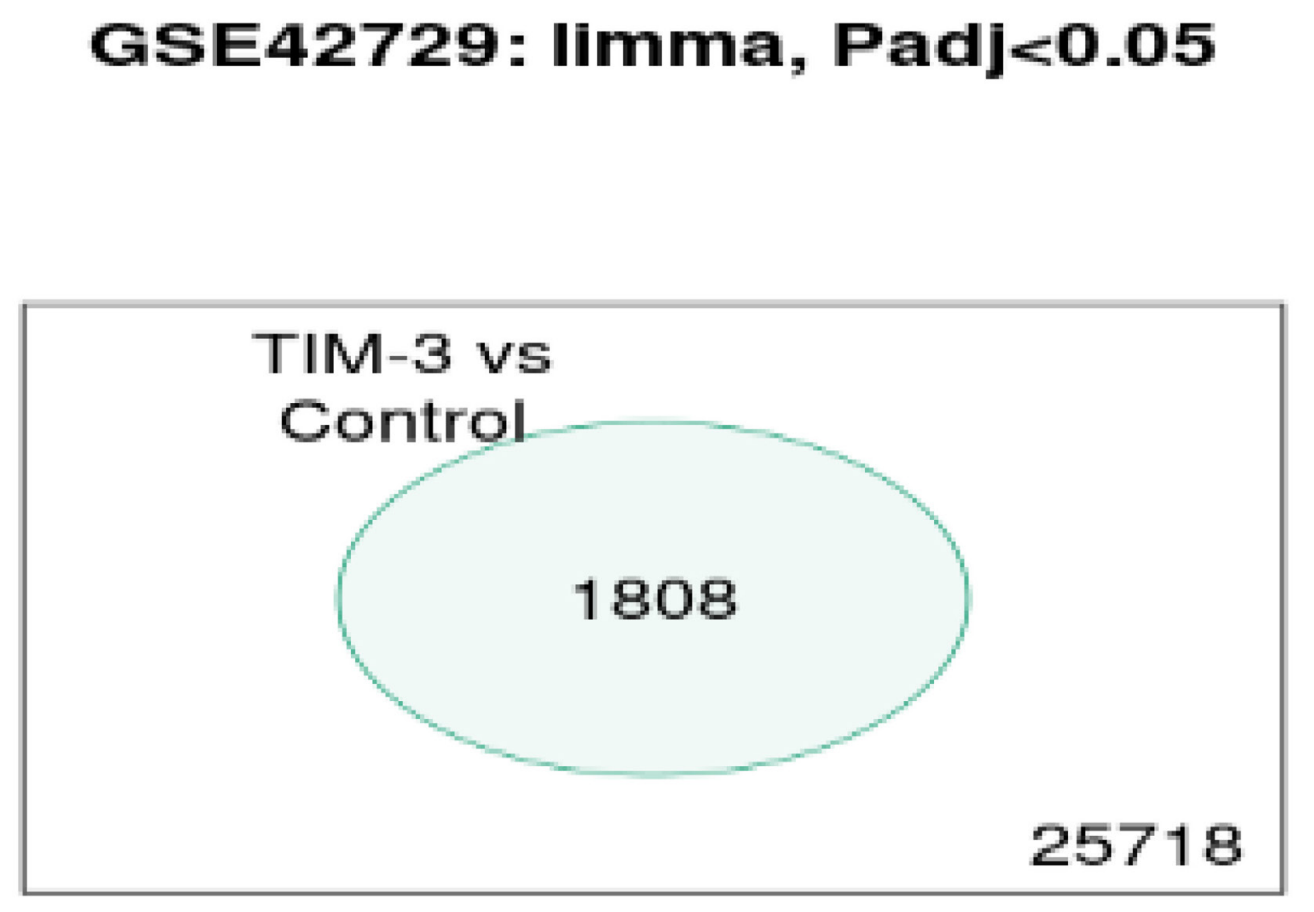
Venn Diagrams of overlapping DEGs.

In reference to Figure 4, the Venn diagram shows that there were a total of 1808 significant genes that were contained in the control and TIM-3 samples.

### Mean-variance Trend

**Figure 5:**
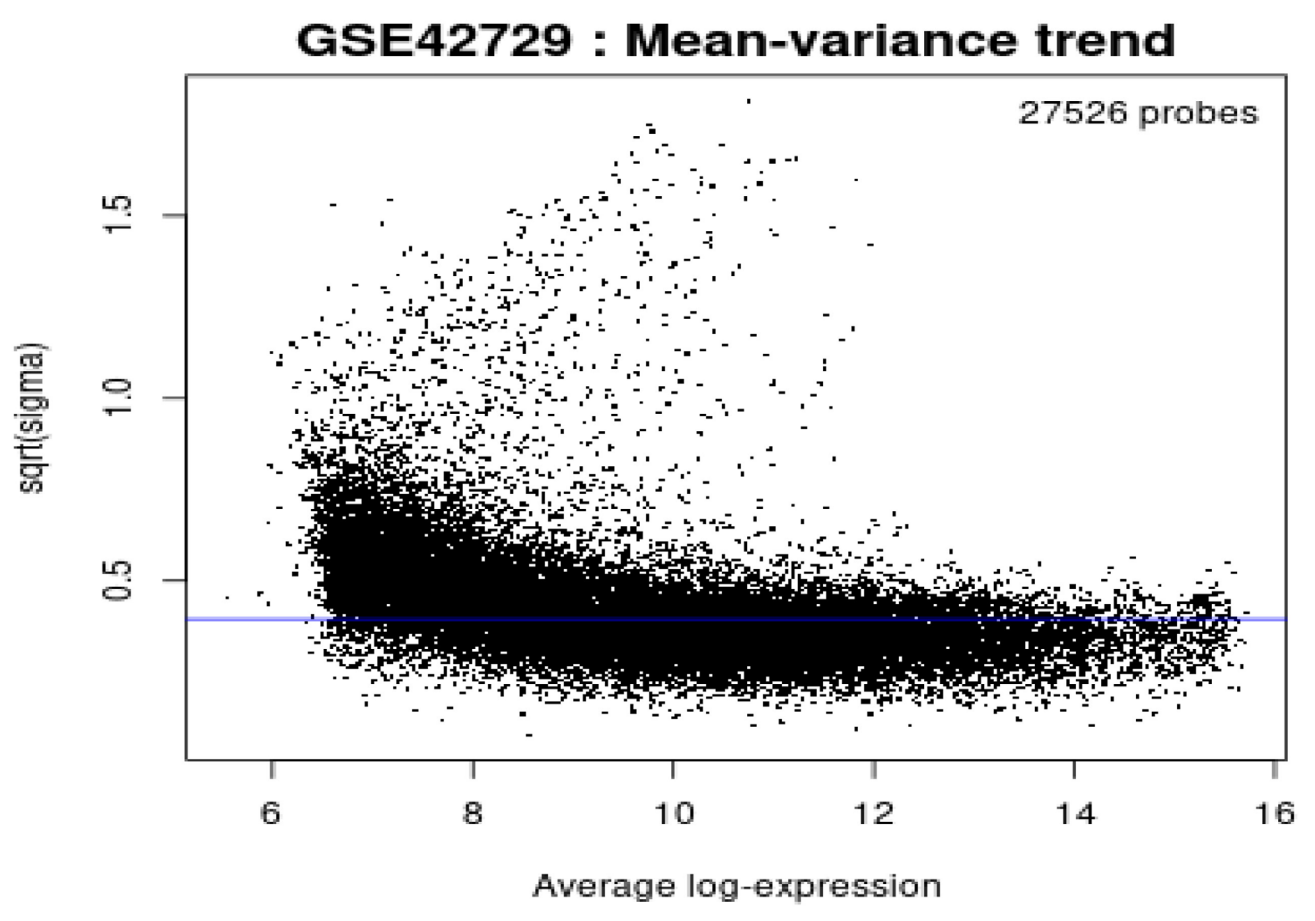
Mean variance trend.

In reference to Figure 5, the mean variance trend was used to explore the gene expression values after fitting the data onto a linear model. There was minimal variation in the data with each point indicating a gene. The blue line represents an estimate of the constant variations.

### Heatmap

**Figure 6:**
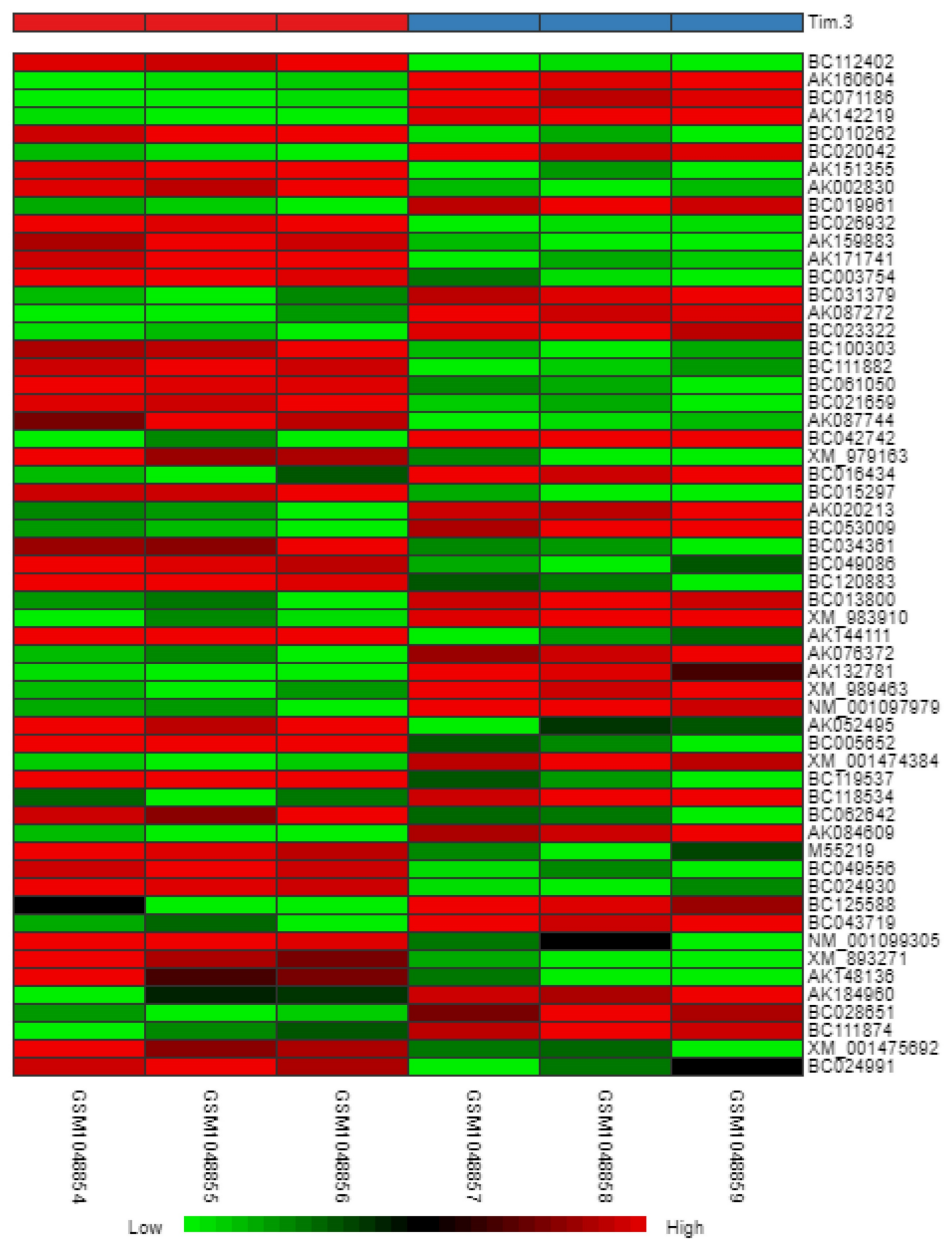
Heatmap of DEGs.

In reference to Figure 6, we obtained a heatmap to show the variations in intensity and changes in expression levels of the DEGs. The map shows samples (columns) and genes (rows) with light green representing smaller changes in the gene expression levels and dark red representing larger changes in gene expression levels.

### PPI Networks

**Figure 7:**
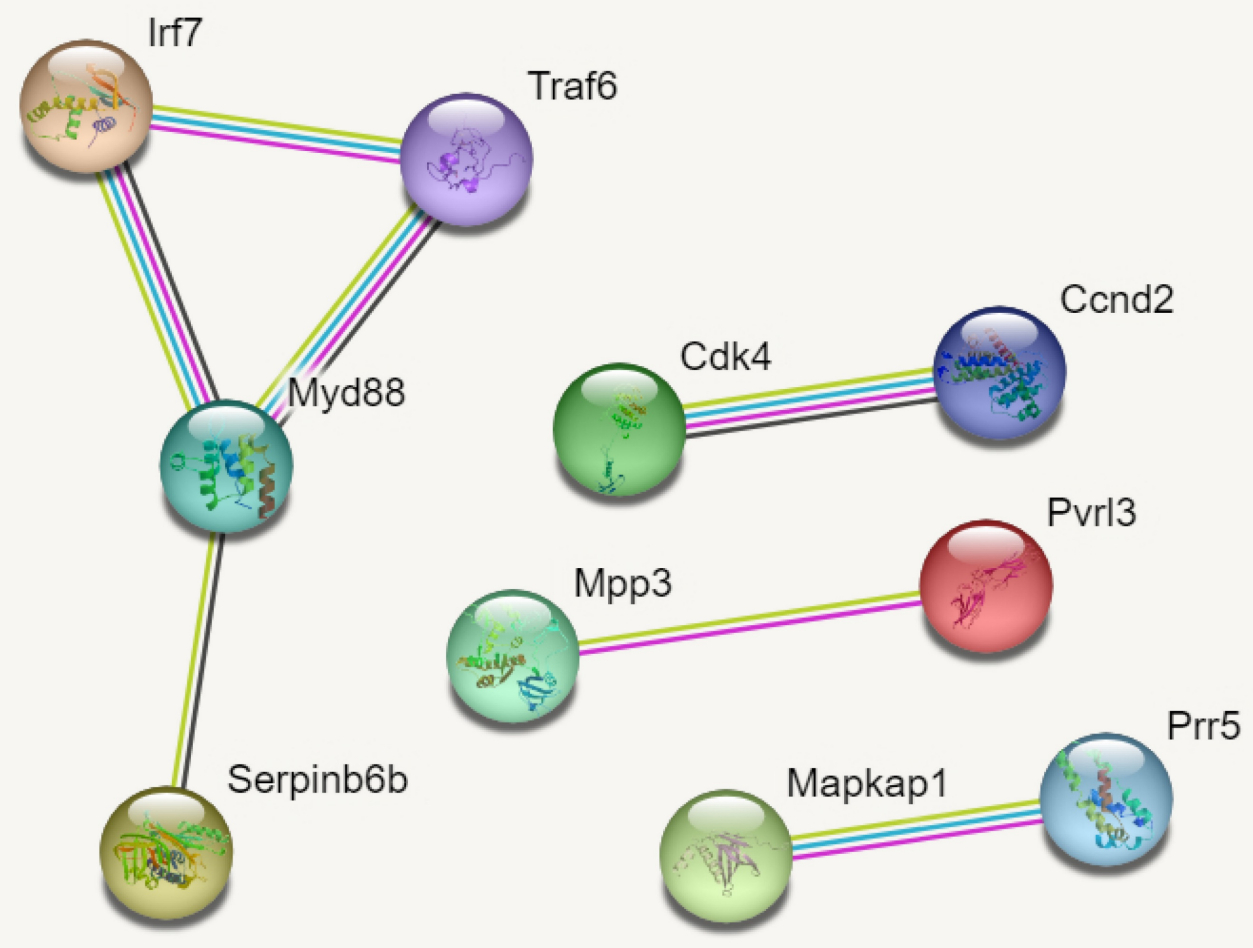
PPI Networks.

In reference to figure 7, we identified 10 hub genes. The PPI network consisted of 10 nodes, 7 edges and an average node degree of 1.4. The average local clustering coefficient was 0.933 with a PPI enrichment p-value of 0.00136, implying significant interactions.

In Figure 7, MYD88 (Myeloid differentiation primary response 88) is an adapter protein involved in the Toll-like receptor (TLR) signalling pathway. TRAF6 (TNF receptor-associated factor 6) is an adapter protein involved in multiple signalling pathways. IRF7 (Interferon regulatory factor 7) is a transcription factor involved in the regulation of the immune response, particularly the production of type I interferons. CDK4 (Cyclin-dependent kinase 4) is a kinase involved in cell cycle regulation. It forms complexes with cyclin D proteins and promotes cell cycle progression. CCND2 (Cyclin D2) is a member of the cyclin D family, which forms complexes with CDK4 and CDK6 to drive cell cycle progression.

MAPKAP1 (Mitogen-activated protein kinase-associated protein 1) is involved in the regulation of the MAPK signalling pathway, which plays a crucial role in cell growth, differentiation, and survival. RICTOR (Rapamycin-insensitive companion of mTOR) is a component of the mTOR complex 2 (mTORC2) and plays a role in cell growth and survival signalling pathways. PRR5 (Proline-rich protein 5) is a protein involved in the regulation of mTOR signalling pathway activity. MPP3 (Membrane protein, palmitoylated 3) is involved in cell adhesion and protein trafficking. SERPINB6B (Serpin Family B Member 6B) is a member of the serine protease inhibitor (serpin) family. PVRL3 (Poliovirus receptor-related 3) is a cell adhesion molecule that is involved in intercellular interactions.

### KEGG Pathways

We identified four significant KEGG pathways that were implicated in digestive tumor immunotherapy and TIM-3; pathways of pancreatic cancer, NF-Kappa B signaling pathway, Toll-like receptor signaling pathway and C-type lectin receptor signaling pathway. NF-kappa B signalling pathway observed in 15 out of 99 networks with a strength of 0.57 and an FDR of 0.0128. Toll-like receptor signalling pathway observed in 14 out of 98 networks with a strength of 0.54 and an FDR of 0.0128.

### Biological Processes

**Figure 9:**
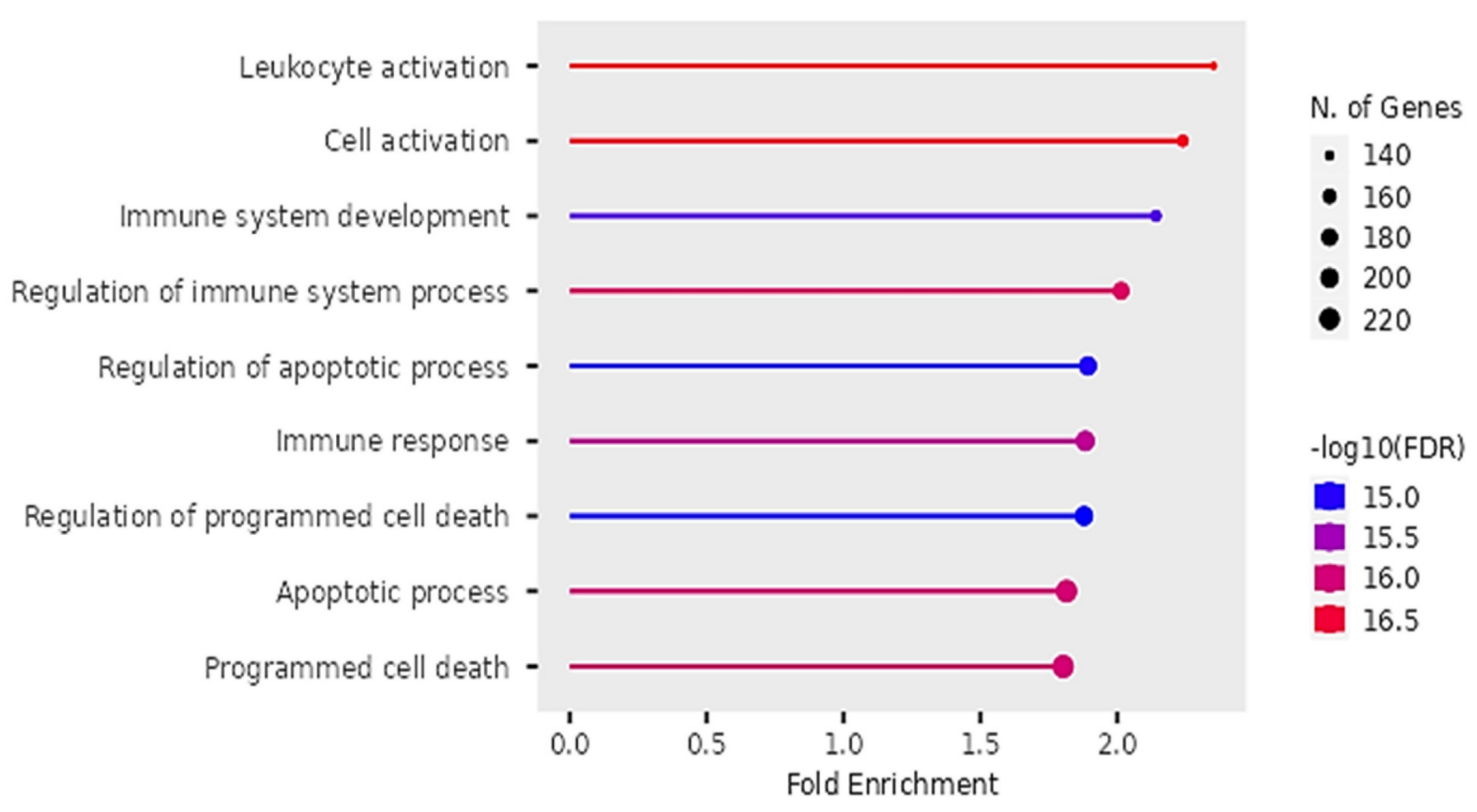
Biological pathways of DEGs identified by Gene Ontology.

Our findings on GO showed that the DEGs were significantly enriched in biological pathways of activating leukocytes, cells, development of the immune system, regulating immune system processes, regulating apoptotic cell processes, immune response and regulation of programmed cell death (see Figure 9).

### Cellular Functions

**Figure 10:**
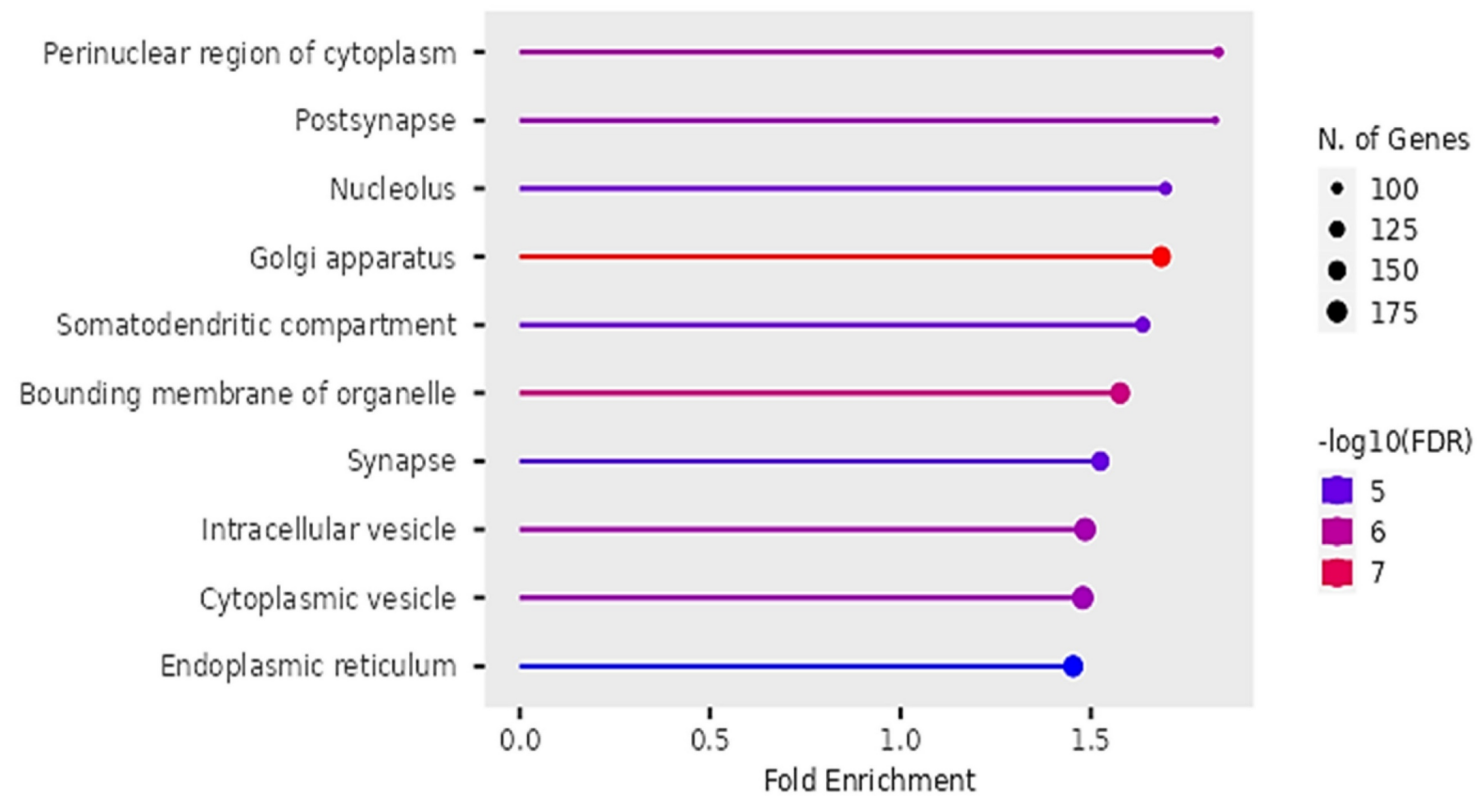
Cellular functions of DEGs identified by Gene Ontology.

According to Figure 10, the differentially expressed genes were enriched in cellular functions of perinuclear region of the cytoplasm, post-synapse, nucleolus, Golgi apparatus, somatodendritic compartments, bounding membrane of organelles, intracellular vesicles, cytoplasmic vesicles and endoplasmic reticulum.

### Molecular Functions

**Figure 11:**
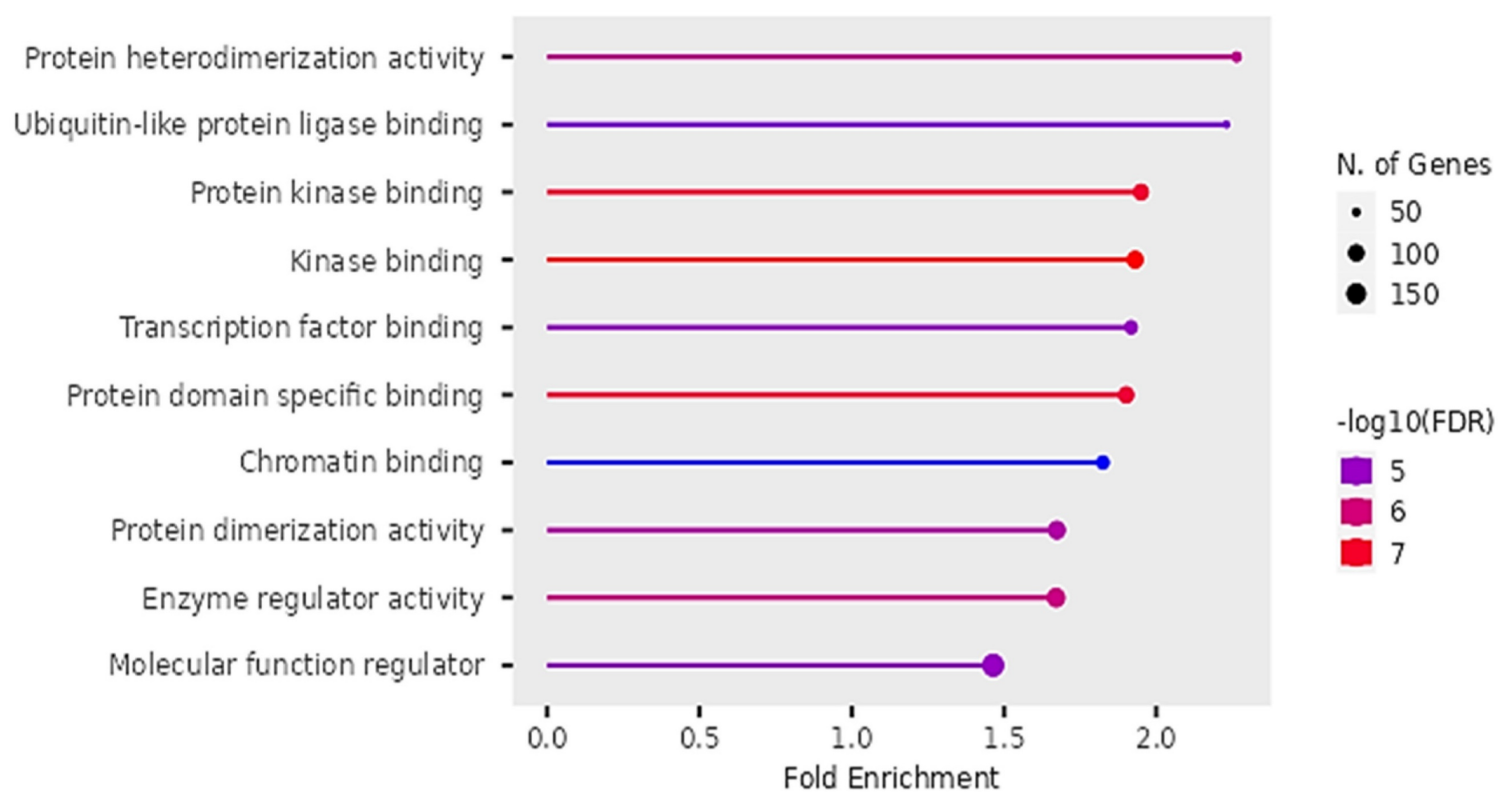
Molecular functions of DEGs identified by Gene Ontology.

According to Figure 11, the differentially expressed genes were enriched in protein heterodimerization, Ubiquitin-like protein ligases, binding of protein kinases, transcription factors, protein domains, chromatin, actions of enzyme regulation and protein dimerization.

## Discussion

Our findings proposed that the significant differentially expressed genes were enriched in biological pathways of activating leukocytes, cells, development of the immune system, regulation of the immune system processes, regulation of apoptotic processes, and programmed cell death. Additionally, we identified four significant KEGG pathways that were implicated in digestive tumor immunotherapy and TIM-3; pathways of pancreatic cancer, NF-Kappa B signaling pathway, Toll-like receptor signaling pathway and C-type lectin receptor signaling pathway.

The findings on Gene ontology of cellular functions were enriched in perinuclear region of cytoplasm, post synapse, nucleolus, Bounding membranes of organelles, synapse, intracellular vesicle, cytoplasmic vesicle, Golgi apparatus and somatodendritic compartments. Moreover, gene ontology revealed that molecular functions were enriched in heterodimerization of proteins, binding of protein kinases, transcription factors, chromatin, protein-domain specific, enzyme regulation and dimerization of proteins. The PPI networks identified 10 hub genes that were implicated in digestive tumor immunotherapy target TIM-3 (MYD88, TRAF6, IRF7, CDK4, CCND2, MAPKAP1, RICTOR, PRR5, MPP3, SERPINB6B AND PVRL3).

TIM-3 is commonly expressed on CD8+ T cells and is a critical sign of malfunctioning of T cells. Anti-tumor activity is enhanced by the loss of TIM-3 on dendrites because it inhibits the dendritic cells from performing regulatory functions while facilitating the functions of CD8+ effector and stem like T-cells 23. Furthermore, the elimination of TIM-3 in dendritic cells has an overall effect of increasing the buildup of reactive oxygen species and activation of the inflammatory cytokines. We observed that the biological pathway of activating leukocytes was implicated in the action of TIM-3 in the development of digestive tumors. The interaction between leukocytes and platelets has a crucial role in the development and progression of cancer.

Stoiber and Assinger 24 suggested that platelets have a critical role in regulating inflammatory cytokines despite their fundamental roles in homeostasis. Leukocytes are actively involved in controlling the immune system through direct and indirect interactions with platelets to enhance the tumor microenvironment. The combination of platelets and leukocytes alter the processes of inflammatory cytokines in various stages of cancer metastasis; for instance, the status of the endothelial cells and recruitment of cells into the tumor sites and microenvironment. Furthermore, the alterations in the activation of platelets leads to enhancement of the coagulation cascades and increased risk in thrombosis.

According to Kral et al. 25 platelets are often activated upon injury to cells and tissues through their TLRs enhancing the production of growth factors and immunomodulation. These intertwined processes promote tumor sculpting and secretion of ADP which promotes the processes of degranulation and release of pro-tumorigenic factors 26. Platelets are often bound to tumor cells through the C-type lectin-like immune receptor 2 pathway that includes interaction with podoplanin expressed on several digestive tumors and is positively associated with poor prognosis. The utilization of the C-type lectin-like immune receptor 2 pathway increases the progression and development of tumors, thrombosis and metastasis of cancer cells. Moreover, interactions between podoplanin and C-type lectin-like immune receptor 2 pathway exacerbates immunosuppression within the tumor microenvironment that increases the rate of growth and development of digestive tumors 27.

Yu et al. 28 suggested that the production of high-mobility group box 1 (HMGB1) by dead cancer cells undergo significant interactions with TLR4 situated on the platelets to regulate the processes of platelet-tumor cell interactions that aids metastasis. Furthermore, the presence of the extracellular matrix after exposure to tumor cells increases the rate of tumor invasion and direct interaction of circulating cancer cells with platelets. These interactions have a significant effect on activating platelets and producing proangiogenic and adhesive cancer components 29.

Platelets and leukocytes often complement each other in the tumor microenvironment just like various type of immune cells that are recruited at the sites of action in tumors. The recruitment of leukocytes and platelets is necessitated in response to continuous cross-interactions between immune cells and tumors mediated by the interplay of host protection and sculpting tumors 30. We propose that the presence of myeloid cells have a crucial role in controlling immune functions because they remove aberrant tissues and cells while releasing the acquired antigens onto the T cells. Myeloid cells consist of several phenotypes that exerts effector cytotoxic lymphocytes and controlling T cells that regulates immune response in various tumors. Furthermore, myeloid cells are also implicated in the regulation of growth of tissues, remodeling of tissues and homeostasis through chemokines, cytokines, scavenging receptors and growth factors. Kral et al. 25 found that platelets enhance the functions of myeloid cells by increasing extravasation of leukocytes and controlling the action of inflammatory cytokines.

The biological pathway of immune system development and regulating immune system were critical in the development of gastric cancers. These findings were consistent with Wang et al. 31 who proposed that the immune system and immune cells are critical in regulating malignant gastric cancers. The immune system is responsible for detecting and eliminating tumor cells. Tumor mutations due to specific and associated antigens occur in the microenvironment and are eliminated by dendritic cells through endocytosis. These antigens are processed as peptides and assembled on major-histocompatibility complexes (MHC) through the endosomes. The action of dendritic cells on the immune system requires an activation signal, for example, the PAMP or DAMP to increase the rate of maturation and subsequent expression of these peptide MHC levels.

Activation of dendritic cells can alter the chemokine receptors and expression of adhesion molecules which increases their response to cytokines and chemokines produced by the tumor draining lymph nodes. The migration of dendritic cells to the draining lymph nodes increases the levels of associated and specific antigens on the CD8+ T cells and CD4+ T cells to initiate an antigen-specific T cell response 32. The successful activation of cytotoxic T cells is aided by dual signals of T-cell receptors and presentation of antigens that are bound to class I molecules or a co-stimulation of CD80 and CD28 molecules. Therefore, activated T cells increases the rate of proliferation and production of memory T cells in response to various pathogens such as cancer. The effector and memory cells then migrate from the draining lymph nodes into the tumors and release cognate antigens in the presence of Th1 cells.

In the effector phase of the immune system, the T cells are responsible for infiltrating the cancer lesions in response to various cytokines and chemokines. The action of infiltrating T cells destroys the tumor cells through direct and indirect approaches. In the direct approach, perforins and granzymes are used to destroy cancer cells. The destruction of cancer cells is a multistep process that involves recognition of antigens, their presentation and effect on tumors. The tumor unique identification of cytotoxic T cells of cognate antigens, increases the activation and creation of immune synapses at the recognition sites. Similarly, these cytotoxic T cells are responsible for the translocation of cytotoxic granules to the immune synapses where they interact with the cell membranes. Perforin undergoes polymerization before insertion into the cell membranes of tumors to create pores and permit the action of granzyme B into the cytoplasm that is responsible for inducing apoptosis. This process has several indirect effects such as production of inflammatory cytokines such as tumor necrosis factor and interferon gamma 33. Once the tumor cells are eliminated by the immune system, survived CD8 positive T cells undergo further differentiation to produce memory cells that are capable of retaining their anti-cancer action and have a robust and quick immune response to tumors.

The biological pathway of regulating apoptotic processes was critical in the growth, development and progression of digestive tumours. Apoptosis is a programmed cell death that maintains tissue homeostasis by removing abnormal and dead tissues 34. A dysregulation of this pathway has been implicated in tumour development and metastasis. Programmed cell death involves several processes and mechanisms commencing with pro-apoptotic proteins to anti-apoptotic proteins that undergo a series of complex interactions to alter the structure of cells to undergo apoptosis. Reduction in apoptosis is positively linked with an increase in the survival of cells and positive outcomes in patients with digestive tumours.

The Bcl-2 family of proteins plays an essential role in regulating apoptosis because it consists of the pro-apoptotic proteins (Bax and Bak) and anti-apoptotic proteins (Bcl-2 and Bcl-xL). An effective balance should be maintained between these proteins to increase the survivability of cells 35. Moreover, the regulation of apoptosis is affected by signaling pathways such as p53 that modulates the expression levels and activity of several apoptotic proteins and altering the sensitivity of cells to apoptosis. Besides, apoptotic proteins, the tumour microenvironment and presence of immune cells may enhance the process of apoptosis. For instance, Cytotoxic T cells are capable of inducing apoptosis by releasing apoptotic molecules.

The KEGG pathway of pancreatic cancer suggested essential molecular processes implicated in the growth and development of pancreatic cancer. This pathway consists of genetic mutations and dysregulation of essential cellular processes. Moreover, this pathway modulates other pathways such as the Notch signaling pathway, P13 and MAPK signaling pathways 36. All these pathways have an integrated role in the proliferation of cells, translocation of cells, and survival of cells in pancreatic cancer. TIM-3 is commonly expressed in tumor infiltrating lymphocytes and is positively associated with dysfunctioning of the immune system leading to immune evasion by tumours. TIM-3 has a negative role in controlling immune responses and immunosuppression within the tumour microenvironment. High expression levels of TIM-3 have been linked with poor prognosis and reduce overall survival in patients of pancreatic cancer.

TIM-3 undergoes significant interactions with its ligands, for example, galectin-9 to initiate inhibitory signals that limit the action of immune cells and the immune system 37. A compromised immune system impairs the action of cytotoxic T cells and lowers the release of pro-inflammatory cytokines leading to an increase in tumour metastasis. Therefore, we propose that TIM-3 has a critical role in immunosuppression and it should be targeted as a novel therapeutic mechanism in the treatment of digestive tumours such as pancreatic cancer. Blocking of TIM-3 by utilizing monoclonal antibodies could be a significant step in clinical research aimed at restoring anti-tumor response and efficacy of immunotherapy.

The KEGG pathway of NF-Kappa B signaling is associated with regulating NF-Kappa B which is a crucial transcription factor in various cellular processes and mechanisms such as cell proliferation, cell survival and inflammatory responses. The pathway consists of complex processes that activates NF-Kappa B in the presence of extracellular stimulus such as stress or pathogens. In normal cell conditions, NF-Kappa B is sequestrated in the cytoplasm due to its interactions with I-Kappa B proteins 38. Once it is activated, it initiates phosphorylation and degradation of I-Kappa B proteins allowing NF-Kappa B to migrate into the nucleus and initiate processes of immune response, cell survival and proliferation. An aberrant activation of this pathway has been implicated in the development of pancreatic cancer, colorectal cancer or gastric cancer.

Our findings propose that dysregulation of this pathway enhances resistance to therapy, invasion and growth of tumours due to increase in cytokines and anti-apoptotic proteins. There is minimal research on the action of TIM-3 on NF-Kappa B signaling pathway. However, TIM-3 has a key role in regulating immune responses and modulating the tumor microenvironment. The interactions between TIM-3 and galectin have inhibitory effects on dendritic cells leading to dampened immune responses. Despite the lack of existence of direct effects between NF-Kappa B and TIM-3, our study suggests that regulation of immune responses by TIM-3 could have potential indirect effects on the NF-Kappa B signaling pathway.

The TLR signaling pathway has an important role in the detection of numerous PAMPs and initiating various response in the immune system. The expression of TLRs on macrophages, B cells or dendritic cells increases their rate of recognizing pathogens 39. TLRs bind to various ligands and increases the recruitment of adapter proteins (for example, Myd88, TRAF6 or TRAM) that controls downstream signaling cascades. These cascades have a ripple effect of activating several transcription factors (for example, AP-1 or NF-Kappa B) and increasing the production of pro-inflammatory cytokines. The CLR signaling pathway consists of C-type lectin receptors that are critical in the recognition of carbohydrates on antigens and pathogens 40. C-type lectin receptors are expressed on natural killer cells, macrophages and dendritic cells. CLR binds to various ligands and activates transcription factors and release of inflammatory cytokines. A dysregulation of TLR and CLR affects the functioning of the immune cells and presentation of antigens within digestive tumours.

Our analysis of PPI networks identified 11 hub genes (Myd88, Traf6, Irf7, Cdk4, Ccnd2, Mapkap1, Prr5, Mpp3, Serpinb6b and Pvrl3) that were implicated in digestive tumours. A dysregulation of the MyD88 gene and TLR signaling can contribute to chronic inflammation and immune activation within the tumor microenvironment. Aberrant activation of the TLR-MyD88 pathway has been observed in various digestive cancers, including colorectal cancer, gastric cancer, and pancreatic cancer. It can promote tumor growth, invasion, and metastasis through the induction of pro-inflammatory cytokines and the modulation of the immune response. The TRAF6 gene encodes the TRAF6 protein, which serves as an adapter molecule in several signaling pathways. TRAF6 plays a crucial role in the activation of NF-κB and MAPK signaling pathways downstream of various receptors, including TLRs. It is involved in the regulation of pro-inflammatory cytokine production, cell survival, and immune responses.

### Strengths and Limitations

The study exhibits strengths in its comprehensive analysis of gene expression and pathways associated with digestive tumor immunotherapy, identifying key molecular mechanisms and potential therapeutic targets. The integration of bioinformatics approaches and the connection to clinical relevance by proposing TIM-3 as a therapeutic target contribute to the study's credibility. However, limitations include reliance on data from the GEO database, potential biases in data analysis, and a lack of experimental validation, which challenges the generalizability of findings. The study acknowledges the complexity of biological systems and the need for further exploration of interactions, particularly between TIM-3 and the NF-Kappa B signaling pathway. Additionally, the clinical translation of computational findings into practical applications faces challenges that require validation through clinical trials.

## Conclusion

In digestive tumors, TIM-3 is often expressed on tumor-infiltrating lymphocytes, including CD8+ T cells. High expression of TIM-3 is correlated with immune dysfunction, T cell exhaustion, and impaired anti-tumor immune responses. The binding of TIM-3 to its ligands, such as galectin-9, triggers inhibitory signals that dampen immune responses. The interaction between TIM-3 and its ligands within the tumor microenvironment contributes to immune suppression and enables tumor immune escape. The presence of TIM-3 on immune cells can impair effector functions, reduce cytokine production, and promote immune tolerance, allowing tumor cells to evade immune surveillance and promote tumor growth. In summary, our findings should be interpreted with caution when applying to humans because in our analysis we used an animal model which could present significant variations in diagnosis and treatment.

## Figures and Tables

**Figure 8:**
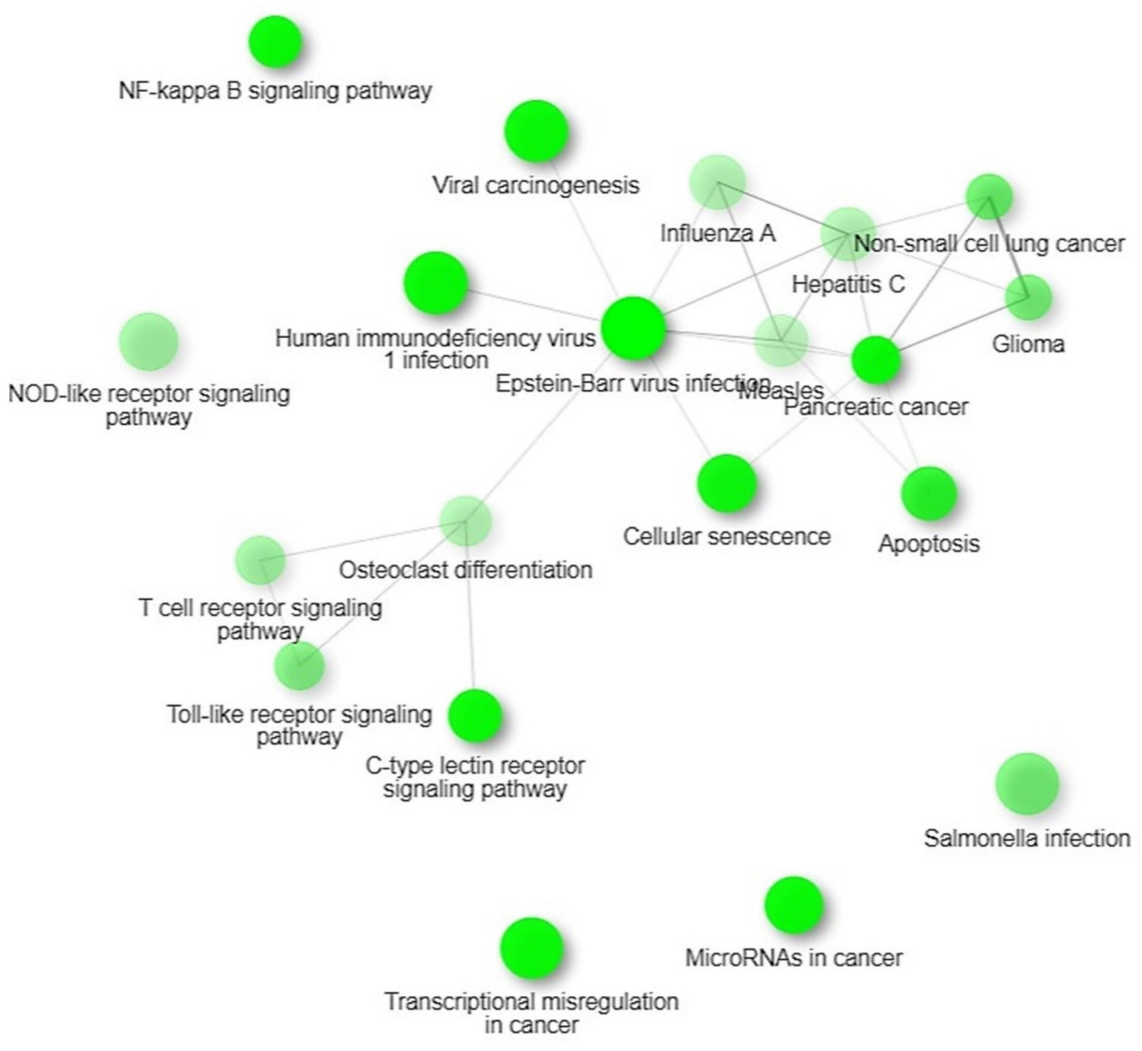
KEGG Pathways.
